# Alcohol Promotes Lipogenesis in Sebocytes—Implications for Acne

**DOI:** 10.3390/cells13040328

**Published:** 2024-02-11

**Authors:** Johannes Kleemann, Jindrich Cinatl, Stephanie Hoffmann, Nadja Zöller, Deniz Özistanbullu, Christos C. Zouboulis, Roland Kaufmann, Stefan Kippenberger

**Affiliations:** 1 Departments of Dermatology, Venereology and Allergy, Goethe University, 60596 Frankfurt am Main, Germany; j.kleemann@med.uni-frankfurt.de (J.K.); n.zoeller@med.uni-frankfurt.de (N.Z.); oezistanbullu@med.uni-frankfurt.de (D.Ö.); kaufmann@em.uni-frankfurt.de (R.K.); 2 Institute of Medical Virology, University Hospital, Goethe University, 60596 Frankfurt am Main, Germany; cinatl@em.uni-frankfurt.de; 3 Dr. Petra Joh-Forschungshaus, 60528 Frankfurt am Main, Germany; 4 Departments of Dermatology, Venereology, Allergy and Immunology, Staedtisches Klinikum Dessau, Brandenburg Medical School Theodor Fontane and Faculty of Health Sciences Brandenburg, 06847 Dessau, Germany; christos.zouboulis@gmx.de

**Keywords:** sebocytes, SZ95 sebocytes, iPSC-derived sebocytes, acne, alcohol abuse, lipogenesis, free fatty ethy esters, orlistat, tetrahydrolipstatin, energy metabolism, seahorse

## Abstract

The oral consumption of alcohol (ethanol) has a long tradition in humans and is an integral part of many cultures. The causal relationship between ethanol consumption and numerous diseases is well known. In addition to the well-described harmful effects on the liver and pancreas, there is also evidence that ethanol abuse triggers pathological skin conditions, including acne. In the present study, we addressed this issue by investigating the effect of ethanol on the energy metabolism in human SZ95 sebocytes, with particular focus on qualitative and quantitative lipogenesis. It was found that ethanol is a strong trigger for lipogenesis, with moderate effects on cell proliferation and toxicity. We identified the non-oxidative metabolism of ethanol, which produced fatty acid ethyl esters (FAEEs), as relevant for the lipogenic effect—the oxidative metabolism of ethanol does not contribute to lipogenesis. Correspondingly, using the Seahorse extracellular flux analyzer, we found an inhibition of the mitochondrial oxygen consumption rate as a measure of mitochondrial ATP production by ethanol. The ATP production rate from glycolysis was not affected. These data corroborate that ethanol-induced lipogenesis is independent from oxygen. In sum, our results give a causal explanation for the prevalence of acne in heavy drinkers, confirming that alcoholism should be considered as a systemic disease. Moreover, the identification of key factors driving ethanol-dependent lipogenesis may also be relevant in the treatment of acne vulgaris.

## 1. Introduction

Our primate ancestors, who lived 10 million years ago, had probably already developed the ability to metabolize ethanol. Unlike apes, which feed on fruits they pick directly from trees, the transition to living on the forest floor required our ancestors to also feed on fallen and partly fermented fruits that contained significant amounts of alcohol. In this respect, it seems likely that an alcohol-rich diet promoted genetic adaptations to this new dietary compound [1]. The ability to produce ethanol-containing beverages through intentional fermentation was probably developed about 9000 years ago [2]. The analgesic, disinfectant, and strongly mind-altering effects of ethanol promoted technical and agricultural progress in the production of ethanol-containing beverages [3]. Nowadays, ethanol is consumed worldwide, however, with high variations between different geographic regions. For example, in the region of the Americas, there are only 16.9% lifetime abstainers in the population aged 15 years and older, whereas this group reaches 94.9% for the Eastern Mediterranean region [4]. This difference can be used to estimate the impact of ethanol consumption on human health. 

The WHO estimates that about 3 million deaths (5.3% of all deaths) worldwide are attributable to ethanol consumption. In addition to injuries, e.g., traffic injuries, violence, homicides, and poisonings, the acquirement of potentially lethal infectious diseases such as HIV through unprotected sex is connected with alcohol intake [5]. Along with a risky sexual behavior goes an increased incidence of other life-threatening conditions, such as HBV and HCV infections [6]. 

Among conditions that are directly associated with the consumption of ethanol are cirrhosis of the liver, epilepsy, pancreatitis, hemorrhagic stroke, hypertensive heart disease, cardiomyopathy, myocarditis, endocarditis, ischemic heart disease, and various malignant neoplasms [7,8,9]. In addition to the well-documented negative impact of ethanol on human health, moderate and sensible use is associated with beneficial effects on atherosclerotic disorders and diabetes mellitus [10]. Also, a protective effect on dementia is discussed [11]. 

With regard to human skin, there is evidence of a link between oral ethanol abuse and skin diseases. Particularly, psoriasis and discoid eczema may be triggered by ethanol misuse [12]. Moreover, there is accumulating evidence that ethanol is affecting varieties of sebaceous gland diseases. The prevalence of acne among adult drinkers is estimated at 1.3% [13] to 27% [14]. This observation prompted us to investigate if there is a connection between ethanol and lipogenesis. The present in vitro study shows for the first time that ethanol is a potent inducer of lipogenesis, particularly triggered by mediators of the non-oxidative metabolism. 

## 2. Materials and Methods

### 2.1. Ethics Statement

This study was conducted according to the Declaration of Helsinki Principles and in agreement with the Local Ethics Commission of the faculty of Medicine of the Johann Wolfgang Goethe University (Frankfurt am Main, Germany). The Local Ethics Commission waived the need for consent.

### 2.2. Chemicals

The alcohol dehydrogenase inhibitors 1,10-phenanthroline, dissolved in dimethyl sulfoxide (DMSO) (Merck, Darmstadt, Germany), and 4-methylpyrazole (Fomepizol) [15], dissolved in medium, were purchased from Sigma-Aldrich (Taufkirchen, Germany). Trans−1,2–dichloroethylene (DCE), an inhibitor of cytochrome p450 2E1 [16], dissolved in DMSO and sodium azide, which inhibits catalase [15], dissolved in medium, were from Sigma-Aldrich. FAEE synthase inhibitor tetrahydrolipstatin (Orlistat^®^) [17], dissolved in DMSO, was from Sigma-Aldrich. Palmitoleic acid ethyl ester (Biomol, Hamburg, Germany) and ethyl oleat (Sigma-Aldrich), frequent products of ethanol metabolism [18,19], were both dissolved in ethanol (final concentration < 0.2%). Arachidonic acid to induce lipogenesis in SZ95 sebocytes [20] was from Sigma-Aldrich. Ethanol (Honeywell, Seelze, Germany), 2-propanol, and methanol (Sigma-Aldrich) were diluted in medium to the indicated concentrations. Sorbitol (Sigma-Aldrich) was dissolved in medium.

### 2.3. Cell Culture

The immortalized human sebaceous gland cell line SZ95 (SZ95 sebocytes) [21] was grown in Dulbecco’s modified Eagle’s medium (Invitrogen, Karlsruhe, Germany) supplemented with 5% fetal bovine serum (FBS) and 1% penicillin/streptomycin solution (Biochrom, Berlin, Germany) in a humidified atmosphere containing 7.5% CO_2_ at 37 °C. The medium was changed every third day. Subconfluent cell cultures were enzymatically split by 0.125% trypsin/0.1% ethylenediamine tetraacetic acid solution and then propagated in culture medium, as described above. For experiments, cells between passages 12 and 24 were used. Human iPSC-derived sebocytes (PCi-SEB_CAU) derived from a Caucasian donor were purchased from Phenocell (Grasse, France). PCi-SEB_CAU were seeded at a density of 2.5 × 10^4^ cells/cm^2^ on fibronectin-coated dishes and propagated in PhenoCULT-SEB culture medium composed of DMEM-GlutaMAX/Ham’s F-12 supplemented with FBS, human insulin, rhEGF, hydrocortisone, adenine, cholera toxin B subunit, and TGFβ inhibitor SB431542 in a humidified incubator (37 °C, 5% CO_2_), according to the manufacturer’s instructions. 

### 2.4. DNA Synthesis 

Cells were cultivated in microwell plates at a density of 2 × 10^4^ cells/0.33 cm^2^ and then exposed to an ascending series of ethanol for 24 h. For the last 24 h, cells were pulsed with 5-bromo-2′-deoxyuridine (BrdU). Subsequently, the incorporation rate of BrdU was determined using a commercial enzyme-linked immunosorbent assay kit (Roche, Mannheim, Germany). Briefly, cells were fixed, and immune complexes were formed using peroxidase-coupled BrdU antibodies. A colorimetric reaction with tetramethylbenzidine as a substrate gives rise to a reaction product measured at 450 nm in a scanning multiwell spectrophotometer (ELISA reader, MR 5000, Dynatech, Guernsey, UK).

### 2.5. Cell Lysis

Cell lysis was quantified using the cytotoxicity detection kit (Roche), which is based on the release of lactate dehydrogenase (LDH) from damaged cells. Briefly, cells were seeded out in microwell plates at a density of 2 × 10^4^ cells/0.33 cm^2^ and treated with an ascending series of ethanol at the indicated concentrations for 24 h. Consecutively, the cell-free supernatants were incubated with NAD+, which is reduced by LDH to NADH/H+. In a second step, NADH/H+ reduced yellow tetrazolium salt to a red-coloured formazan salt. The amount of red colour is proportional to the number of lysed cells. For quantitation, the absorbance of the reaction product was measured at 490 nm using a multiwell spectrophotometer. Supernatants treated with Triton X100A served as positive control.

### 2.6. Detection of Lipids

Lipogenesis was displayed qualitatively and quantitatively using Nile red staining, as described [22,23]. Briefly, SZ95 cells were cultured in 48-well microwell plates (1.5 × 10^5^ cells/0.95 cm^2^) and treated with increasing amounts of ethanol (or other alcohols), ranging from 1% to 4%, corresponding to 171 mM to 685 mM for 24 h, and inhibitors were added 1 h before treatment. Qualitative display of lipids was performed according to Schneider and Zouboulis [24]. Briefly, adherent SZ95 cells were washed twice with PBS and then stained with Nile red (Sigma-Aldrich, 1 µg/mL, 20 min, 37 °C). Nuclei were stained with the DNA-binding fluorochrome bisbenzimide (Sigma-Aldrich, 2 mg/mL, 20 min, RT) before observation under a fluorescence microscope (Axiovert, Zeiss, Oberkochen, Germany). Quantitative analysis was performed as described [25]. Briefly, treated cells were first trypsinated, centrifuged (200× *g*), and then incubated with the Nile red solution. After a washing step, PBS cells were resuspended in PBS and instantly analysed by a BD FACScan Cytometer (Becton Dickinson, Franklin Lakes, NJ, USA), with excitation at 485 nm and emission at 565 nm to detect red fluorescence.

### 2.7. Measurement of Cellular Respiration and Mitochondrial Stress

Adenosine triphosphate (ATP) production was measured using the Seahorse XF Real-Time ATP Rate Assay Kit (Agilent, Santa Clara, CA, USA) by following the manufacturer’s instructions. Briefly, SZ95 cells were exposed to different concentrations of ethanol, as indicated. After 7 h, the rate of ATP production from glycolysis and mitochondria was simultaneously quantified using the Seahorse XFe96 Analyzer (Agilent) by determining the oxygen consumption rate (OCR) and extracellular acidification rate (ECAR) in response to injections of oligomycin and rotenone/antimycin A. Moreover, the mitochondrial function was assed using the Seahorse XF Mito Stress Test Kit (Agilent). After treatment with ethanol for 7 h, the basal respiration, ATP-linked respiration, maximal and reserve capacities, and non-mitochondrial respiration were determined using sequential injections with oligomycin, carbonyl cyanide-4 (trifluoromethoxy) phenylhydrazone (FCCP), and rotenone/antimycin A. The data were analysed and exported using the Seahorse Wave Desktop Software 2.6 (Agilent) and further processed using Microsoft Excel (Redmond, WA, USA).

### 2.8. Presentation of Data and Statistical Analysis

All data are presented as mean values ± standard deviation. Statistical significance of the data was calculated by the Wilcoxon–Mann–Whitney U-test (BIAS, Frankfurt, Germany). Each set of data was related to the referring untreated controls. Significant differences are denoted by asterisks; *p*-values are considered significant with *p* < 0.05.

## 3. Results

### 3.1. Ethanol Induces Lipogenesis

**Figure 1:**
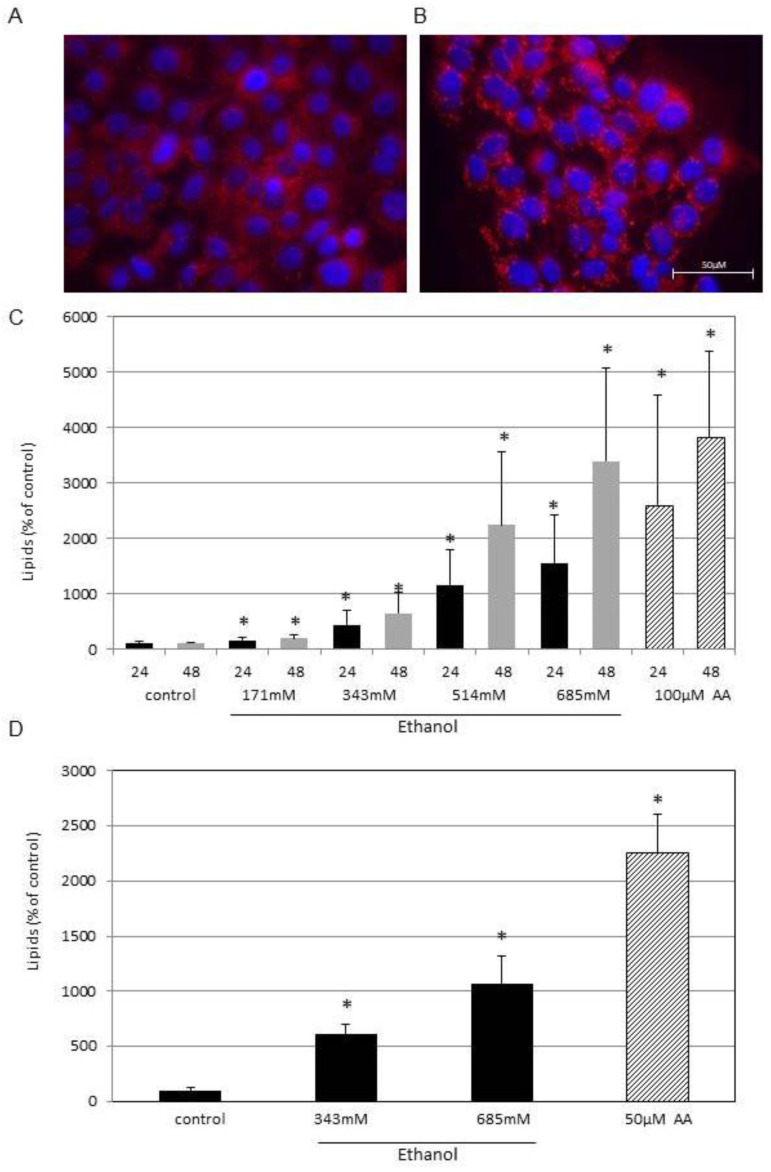
Ethanol induces lipogenesis. SZ95 sebocytes were (**A**) held in regular medium or (**B**) treated with 685 mM ethanol for 24 h. Consecutively, lipids were stained with Nile red solution and displayed by fluorescent microscopy. Nuclei were stained with the DNA-binding fluorochrome bisbenzimide (2 mg/mL, 20 min, RT). Quantitative lipid staining in (**C**) SZ95 cells and (**D**) PCi-SEB_CAU after treatment with ethanol at the indicated concentrations after 24 h (PCi-SEB_CAU) and 24 h and 48 h (SZ95), as measured by FACS. Treatment with arachidonic acid (AA) served as positive control. Each column in (**C**) represents the mean of 28 experiments and in (**D**) of 4 experiments. The standard deviations are indicated. Datasets were statistically compared to the controls. * *p* < 0.05.

In order to test whether ethanol has an effect on sebocyte lipogenesis, SZ95 cells were treated with different concentrations of ethanol for 24 h or 48 h. Consecutively, cells were stained with Nile red solution. The accumulation of lipids after 24 h in the presence of 685 mM ethanol is shown through fluorescent microscopy (Figure 1A,B).

The quantitative results generated by FACS analysis are given in Figure 1C, showing a concentration-dependent induction of lipids. Already, 1% ethanol, equal to 171 mM, yielded a significant increase to 147% after 24 h and 180% after 48 h. In 4% ethanol, equal to 685 mM, which was the highest concentration tested, the lipid-dependent fluorescence increased to 1532% after 24 h and 3390% after 48 h compared to untreated controls. The positive control was 100 mM arachidonic acid, a well-known inductor of lipogenesis [20]. Moreover, the effect of ethanol on lipogenesis was tested in human iPSC-derived sebocytes (PCi-SEB_CAU) (Figure 1D). Similar to experiments performed with SZ95 cells, PCi-SEB_CAU showed a concentration-dependent upregulation of lipogenesis by ethanol. Moreover, it was shown that ethanol induces lipids in adipose-derived stem cells (ADSCs), indicating adipogenic differentiation (Figure S1).

### 3.2. Moderate Effects of Ethanol on DNA Synthesis and Membrane Integrity

**Figure 2:**
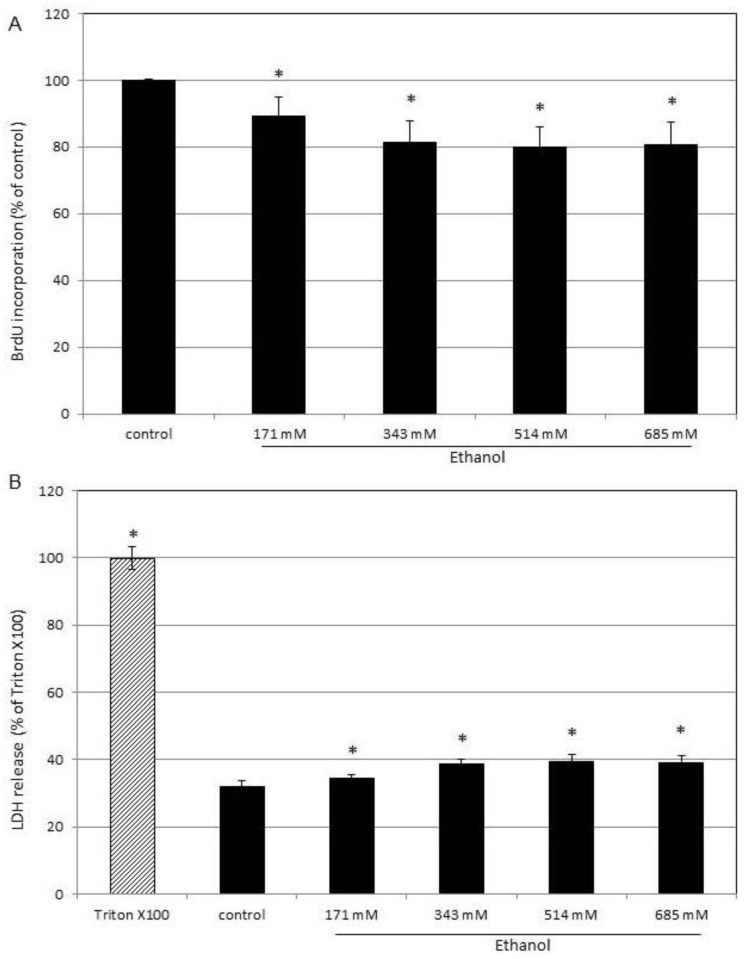
Effect of ethanol on DNA synthesis and LDH release. SZ95 sebocytes were cultured in medium with increasing concentrations of ethanol as indicated. (**A**) After 24 h, the incorporation of 5-bromo-2′-deoxyuridine (BrdU) in the DNA as a measure for DNA synthesis was determined. Cells cultured under standard conditions without ethanol served as control and were set to 100%. (**B**) Cell integrity was monitored after 24 h by assaying LDH activity in supernatants. Complete release of LDH was achieved by treatment with 1% Triton X-100, which served as control and was set to 100%. Each bar represents the mean of 6 independent experiments. Standard deviations are indicated. Data were compared to controls. * *p* < 0.05.

In the following, basic parameters such as DNA synthesis and membrane integrity in response to ethanol were determined in SZ95 sebocytes (Figure 2). 

After 24 h, the DNA synthesis, as determined by BrdU incorporation, decreased in a concentration-dependent manner to 80% at 685 mM (4%) ethanol compared to untreated controls (Figure 2A). Moreover, the release of LDH from cells, as a measure of membrane integrity, was determined (Figure 2B). The basic level of LDH in cell supernatants of untreated controls was 32% compared to Triton X100-treated cells, in which LDH was completely released. In cells treated with increasing amounts of ethanol for 24 h, the LDH level increased to 39% at 685 mM (4%) ethanol.

### 3.3. Effect of Other Alcohols on Lipogenesis

**Figure 3:**
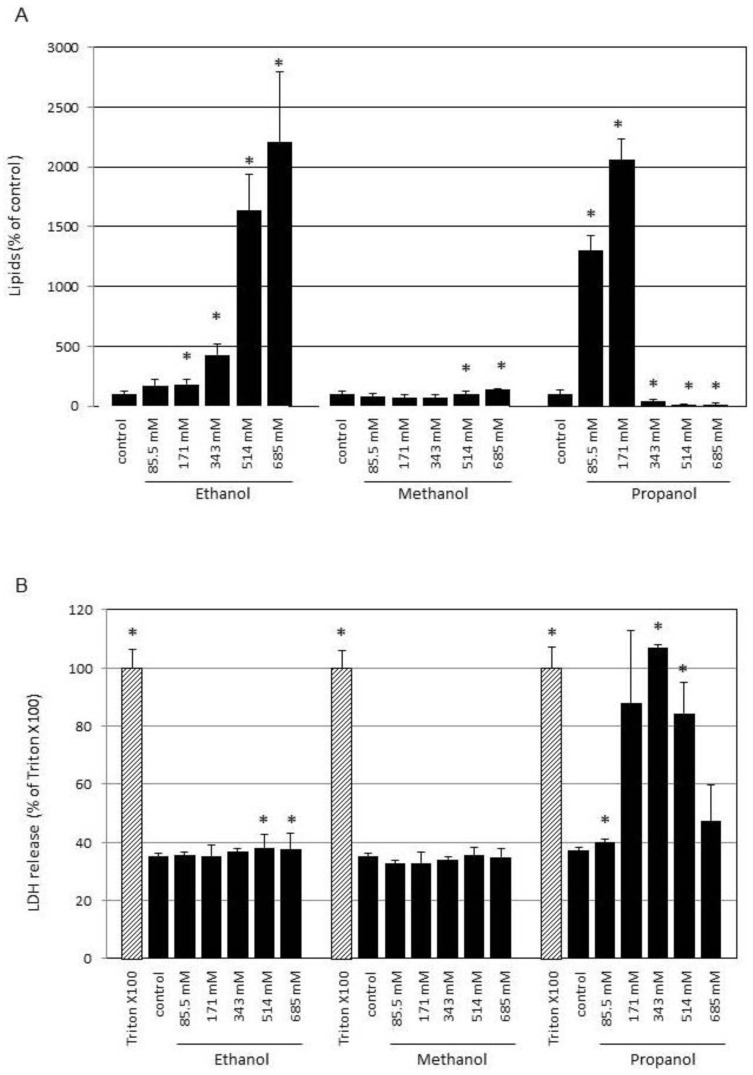
Effect of different alcohols on lipogenesis and LDH release. SZ95 sebocytes were treated with 85.5 mM, 171 mM, 343 mM, 514 mM and 685 mM ethanol, methanol or propanol for 24 h. In (**A**) the results of quantitative lipid staining are shown. Cells cultured under standard conditions without any alcohol served as control and were set to 100%. (**B**) The effect of different ethanol, methanol and propanol on cell integrity was monitored after 24 h by assaying LDH activity in supernatants. Complete release of LDH was achieved by treatment with 1% Triton X-100 which served as control and was set to 100%. Each bar represents the mean of 6 independent experiments. Standard deviations are indicated. Data were compared to controls. * *p* < 0.05.

To test whether the lipogenetic effect of ethanol is specific, other alcohols were tested at the same concentration (Figure 3A). Treatment with increasing concentrations of methanol showed no significant effect on lipogenesis in SZ95 sebocytes. In contrast, treatment with propanol at 85.5 mM already showed a significant increase to 1298% compared to untreated controls. Lipogenesis further increased to 2059% by treatment with 171 mM. No lipogenesis was detected at higher concentrations. For methanol, no increase in LDH release, as a measure of membrane damage, was detected at the concentrations tested (Figure 3B). In contrast, treatment with propanol resulted in a jump in LDH release at 171 mM propanol, which was within the range of complete LDH release induced by Triton X100 (Figure 3B). At the highest propanol concentration tested (685 mM), the detected LDH level in the cell supernatant decreased again. This may be due to early and complete cell disruption with advanced enzyme degradation.

### 3.4. Non-Oxidative Metabolism of Ethanol (NOME) Triggers Lipogenesis

**Figure 4:**
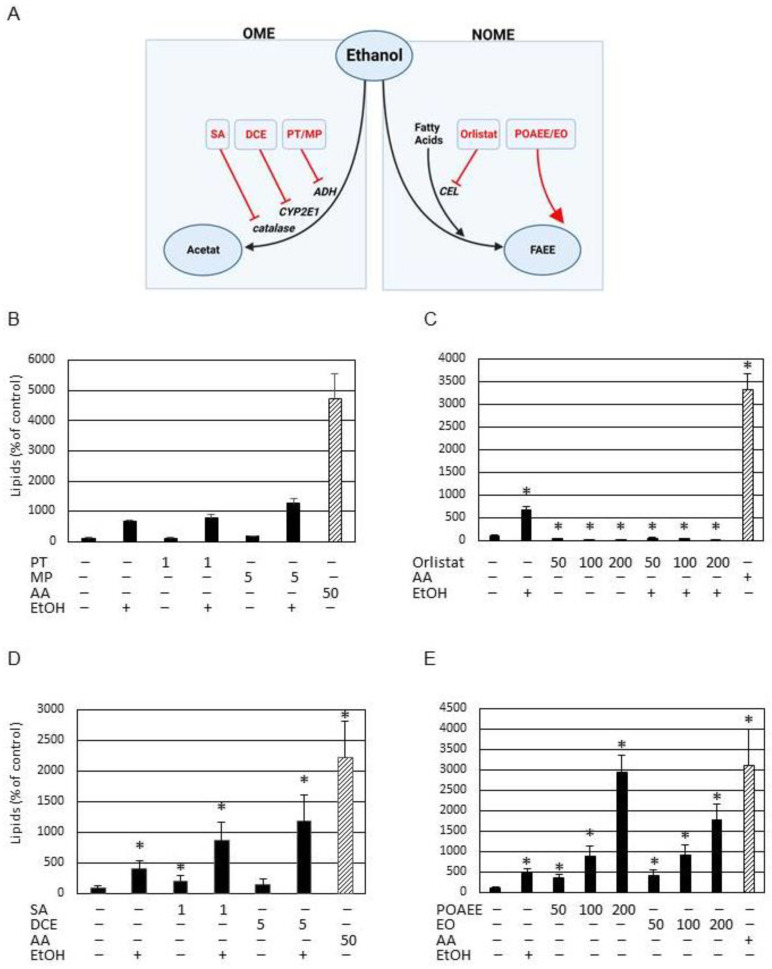
Differentiation between the effect of NOME and OME on SZ95 sebocyte lipogenesis. (**A**) Schematic presentation of ethanol metabolism with regard to OME (oxidative metabolism of ethanol) and NOME (non-oxidative metabolism of ethanol). For OME, the relevant enzymes are ADH (alcohol dehydrogenase), CYP2E1 (cytochrome p450 2E1), and catalase. Specific inhibitors are PT (1,10-phenanthroline), MP (4-methylpyrazole), DCE (trans-1,2-dichloroethylene), and SA (sodium azide). NOME yields in FAEEs (fatty acid ethyl esters), a product of ethanol and fatty acids, catalysed by CEL (carboxy ester lipases). Orlistat (also known as tetrahydrolipstatin) is an inhibitor of CEL. POAEE (palmitoleic acid ethyl ester) and EO (ethyl oleate) are typical FAEEs produced by NOME. Quantitative ethanol-induced lipogenesis in the presence of (**B**) ADH inhibitors PT or MP, (**C**) CEL inhibitor Orlistat and (**D**) CYP2E1 and catalase inhibitors DCE or SA. (**E**) Quantitative lipogenesis in SZ95 cells measured after the addition of the FAEE POAEE or EO. AA (arachidonic acid) served as positive control, EtOH (ethanol). Each bar represents the mean of 4 independent experiments. Standard deviations are indicated. Data were compared to untreated controls. * *p* < 0.05.

The above-presented data show an induction of lipogenesis by ethanol in SZ95 sebocytes. Next, it was examined if specific metabolites of ethanol are responsible for the observed effect. Figure 4A shows a scheme of the ethanol metabolism with specific regard to the oxidative metabolism of ethanol (OME) and the non-oxidative metabolism of ethanol (NOME). Blocking the enzyme alcohol dehydrogenase (ADH) by 1,10-phenanthroline (PT), an inhibitor of ADH class I, II, and III or 4-methylpyrazole (MP), an inhibitor of ADH class I and II [15], did not reduce ethanol-induced lipogenesis (Figure 4B). In contrast, the addition of orlistat (Tetrahydrolipstatin), an inhibitor of carboxy ester lipases [17], led to a complete inhibition of ethanol-induced lipogenesis (Figure 4C). These results show that NOME is relevant for the lipogenic effect of ethanol. Corroboratively, trans-1,2-dichloroethylene (DCE), an inhibitor of cytochrome p450 2E1 (CYP2E1) [16], blocks the oxidative degradation of ethanol. In the presence of DCE, basic and ethanol-induced lipogenesis become amplified (Figure 4D). Likewise, the inhibition of catalase by sodium azide [15] amplified the effect of ethanol on lipogenesis (Figure 4D). These data indicate that OME is not relevant for lipogenesis. In contrast, the addition of palmitoleic acid ethyl ester or ethyl oleate, which are typical fatty acid ethyl esters (FAEEs) formed during NOME [18,19], shows a concentration-dependent induction of lipogenesis (Figure 4E).

### 3.5. Ethanol Inhibits Mitochondrial ATP Production

**Figure 5:**
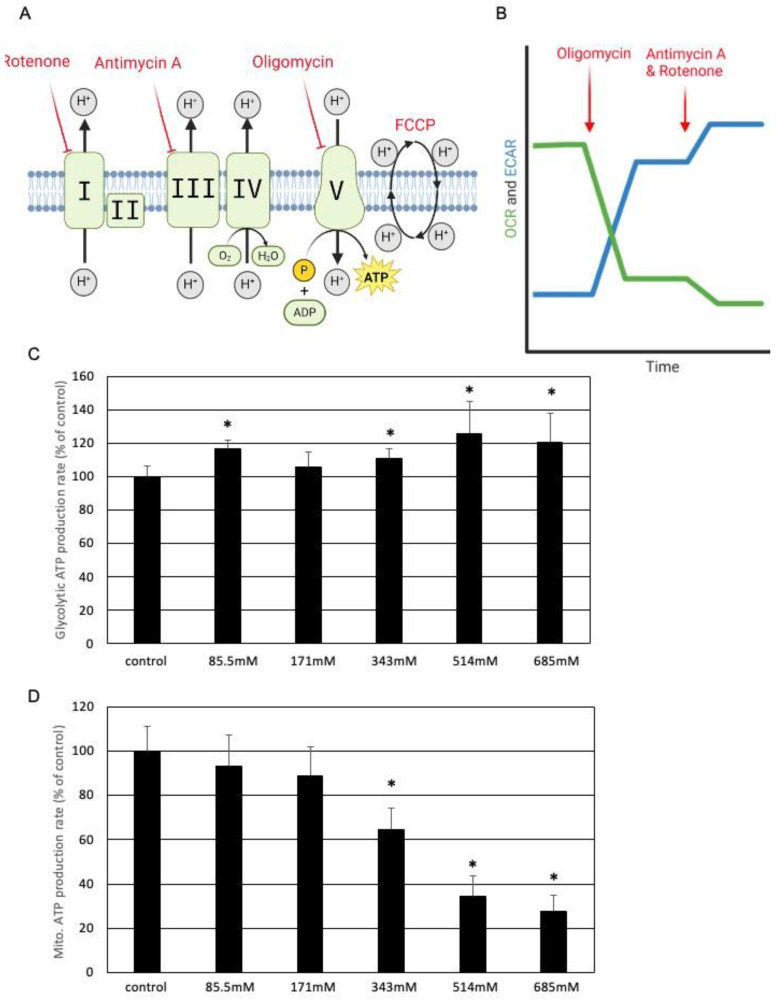
Ethanol inhibits mitochondrial ATP production. (**A**) Representative scheme displaying the targets of different inhibitors of the mitochondrial respiratory chain used. (**B**) Schematic effect of serial application on oligomycin followed by actinomycin A/rotenone on OCR and ECAR. Effect of increasing amounts of ethanol on (**C**) glycolytic and (**D**) mitochondrial ATP production rate in SZ95 sebocytes. Each bar represents the mean of 8 experiments. The standard deviations are indicated. Data were related to the untreated controls. * *p* < 0.05.

In order to test the effect of ethanol on metabolic parameters, the oxygen consumption rate (OCR) and the extracellular acidification rate (ECAR) were measured in real time by using serial injection of inhibitors targeting specific elements of the respiratory chain (Figure 5A). 

**Figure 6:**
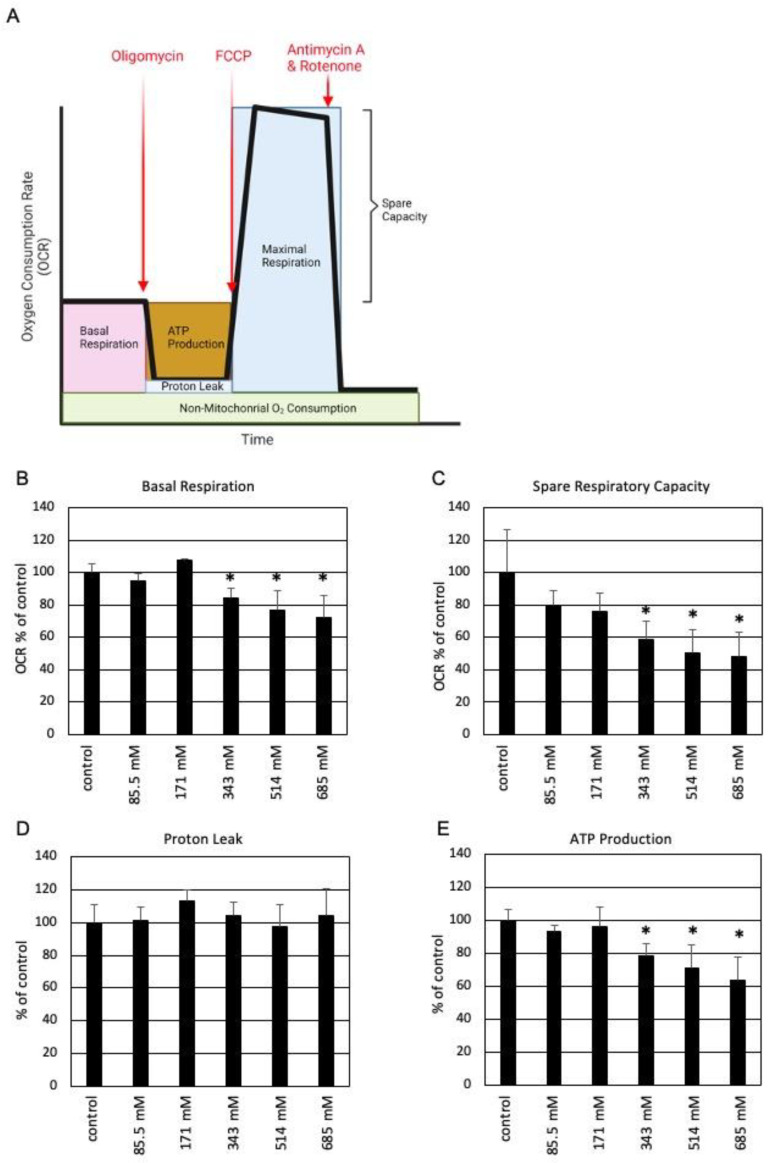
Ethanol inhibits basal respiration, ATP production, and spare respiratory capacity in mitochondria of SZ95 sebocytes. (**A**) Schematic effect of serial application of respiratory chain inhibitors on key parameters of mitochondrial respiration. Concentration-dependent inhibition of (**B**) basal respiration and (**C**) spare respiratory capacity by ethanol. (**D**) Leakage of protons is not increased by ethanol. (**E**) Concentration-dependent decrease in ATP production by ethanol. Each bar represents the mean of 8 experiments. The standard deviations are indicated. Data were related to the untreated controls. * *p* < 0.05.

The effect of sequential injections of oligomycin, antimycin A, and rotenone on OCR and ECAR is schematically shown in Figure 5B. After incubation with the indicated ethanol concentrations for 7 h, the mitochondrial and glycolytic ATP production rate in SZ95 sebocytes was calculated from OCR and ECAR. It was found that the glycolytic ATP production rate was not affected by ethanol, even at high concentrations (Figure 5C). In contrast, a concentration-dependent inhibition of the mitochondrial ATP production rate by ethanol was detected (Figure 5D). Next, the effect of ethanol on mitochondrial function was further specified (Figure 6). Figure 6A schematically displays the effect of serial injections of respiratory chain inhibitors on key parameters of mitochondrial respiration, as measured by OCR. It was found that the basal respiration and the spare respiratory capacity are inhibited by ethanol in a concentration-dependent manner (Figure 6B,C). While the leakage of protons is largely unaffected by ethanol (Figure 6D), ATP production is inhibited (Figure 6E). The latter corroborates findings from above using the Seahorse XF Real-Time ATP Rate Assay Kit (Figure 5D).

## 4. Discussion

In the present study, we show that alcohol (ethanol) is a strong trigger for lipogenesis in SZ95 sebocytes and human iPSC-derived sebocytes. This finding fits with clinical findings found in alcohol abuse, describing pathological skin conditions associated with sebaceous gland disorders, including acne [26]. In particular, we provide evidence that the non-oxidative metabolization of ethanol (NOME) is responsible for the lipogenic effect. In particular, we identified fatty acid ethyl esters (FAEEs), which are esterification products of fatty acids, and alcohol as stimuli for lipogenesis. FAEEs are generated by carboxylester lipase (CEL) [18]. In the presence of tetrahydrolipstatin (Orlistat^®^), a potent inhibitor of CEL [17], the alcohol-induced lipogenesis was completely inhibited, which speaks to the generation of FAEEs in these cells. Corroboratively, the addition of ethyl oleate (EO) and palmitoleic acid ethyl ester (POAEE), which are predominant FAEEs reported in alcoholic patients [18,19], significantly induced lipogenesis. In addition to the generation of FAEEs in sebocytes, there are also indications for an ethyl ester formation within the gastrointestinal tract from ingested ethanol and fatty acids [27]. Therefore, the systemic spreading of these metabolites may also manifest in the skin. In general, FAEEs are considered to be toxic mediators of ethanol-induced organ damage, with high concentrations in the pancreas, liver, and adipose tissue [27]. As FAEEs are hydrophobic, an accumulation in lipid-rich cells such as sebocytes is also likely.

In addition to NOME, the oxidative metabolization of ethanol (OME) produces acetaldehyde, which is then converted to acetate [28]. Among the ethanol-oxidizing enzymes are alcohol dehydrogenases (ADH), cytochrome P450 2E1 (CYP2E1), and catalase [29]. Although ADH families are primarily found in the cytosol of hepatocytes, they are also detected in the skin epidermis and dermis [30]. The function of this class of enzymes in skin is a subject of debate. In addition to the catabolism of systemic alcohol, these enzymes may also contribute to the elimination of ethanol derived from skin microbiota [31]. However, inhibition of ADH by 1,10-phenanthroline (PT) (specific for ADH class I, II, and III) or 4-methylpyrazole (MP) (specific for ADH class I and II) (Bhopale et al., 2014) showed no effect on ethanol-induced lipogenesis. Another enzyme involved in the detoxification of ethanol is CYP2E1, catalysing the reaction from ethanol to acetaldehyde using molecular oxygen similar to ADH [32,33]. Although prototypically expressed in high amounts in the microsomal fraction of liver tissue, CYP2E1 is also detected in human skin [34]. Inhibition of CYP2E1 using trans-1,2-dichloroethylene (DCE) [16] also shows no effect on ethanol-driven lipogenesis. Moreover, the effect of catalase inhibition was tested. Catalase, localized in peroxisomes, also oxidizes ethanol to acetaldehyde in the presence of hydrogen peroxide-producing enzymes such as NADPH oxidase or the xanthine oxidase [29]. Catalase was detected in considerable amounts in the skin [35,36]. Our results show that the inhibition of catalase by sodium azide leads to increased lipogenesis, even without ethanol. The combination of ethanol and sodium azide induced lipogenesis more than ethanol alone. This may be due to the shifting ethanol metabolism from OME to NOME. 

Our data showed that NOME is responsible for the lipogenic effect in sebocytes. This is interesting as most of the per os ingested alcohol is metabolized in the liver by OME, affecting the energy metabolism of the mitochondria [37]. Corroboratively, we found a strong inhibition of mitochondrial ATP production by ethanol, while the ATP production via glycolysis was unaffected. Of note, the major part of ATP production in sebocytes is derived from glycolysis, which may explain the relatively high tolerance of these cells against ethanol.

Similar to ethanol, methanol, a compound known for intentionally provoked intoxications, also becomes oxidized to toxic metabolites (formaldehyde and formic acid) [38]. Although chemically closely related to ethanol, methanol offers no lipogenic effect, which speaks to oxidative metabolization in sebocytes. 

Propanol, typically contained in disinfectant, hand sanitizer, cosmetics, and “rubbing alcohols”, shows a strong lipogenic effect at low concentrations (<171 mM); at higher concentrations, the toxic effect becomes predominant. Systemic intoxication in humans is rare, and most happen non-intentionally in children [39]. An oxidative metabolization by ADH to acetone and other metabolites is described [40]. Whether these metabolites are responsible for the lipogenic effect is an open question. The topical application of propanol for treating acne, as recommended by some internet forums, does not seem to be helpful with regard to the lipogenic effect.

In sum, the present work features a new pathophysiological quality of ethanol on skin sebocytes, which fits well with clinical observations showing a higher prevalence for acne in alcohol abusers. In addition, there is an association with rosacea, a chronic inflammatory skin condition characterized by redness and papules on the face. In a recent meta-analysis, alcohol consumption was shown to be a risk factor for phymatous rosacea, which is characterized by a thickening of the skin, typically on the nose, a condition known as rhinophyma [41]. Clinically, rosacea is considered an inflammatory disease of the sebaceous gland, although the aetiology is largely unknown. Future studies may address the question of whether alcohol triggers at least subclasses of rosacea through the mechanisms presented in this paper.

In addition, experiments with ADSCs suggest that ethanol can trigger the adipogenic transdifferentiation of stromal stem cells (Figure S1). It is possible that this contributes to the body remodelling often seen in alcoholics with abdominal fat accumulation. Interestingly, alcohol is also produced within the human organism. Gut fermentation syndrome (or auto brewery syndrome) describes a condition where the microbial fermentation of carbohydrates in the gastro-intestinal tract leads to increased blood alcohol levels [42]. Moreover, there are indications for the existence of intracellularly synthesized ethanol [43]. Whether alcohol from these sources has an impact on skin conditions such as acne is an interesting question. 

## Data Availability

Data are contained within the article.

## Supplementary Materials

The following supporting information can be downloaded at: https://www.mdpi.com/article/10.3390/cells13040328/s1, Figure S1: Ethanol induces adipogenic differentiation in adipose-derived stem cells (ADSC).
