# Supplementary material for: Alcohol Promotes Lipogenesis in Sebocytes—Implications for Acne
Source: Cells. 2024 Feb 11;13(4):328. doi: 10.3390/cells13040328 (PMC10886960; doi:10.3390/cells13040328)
Supplement: Supplementary file 1 [file cells-13-00328-s001.zip › cells-2841512-supplementary.pdf]

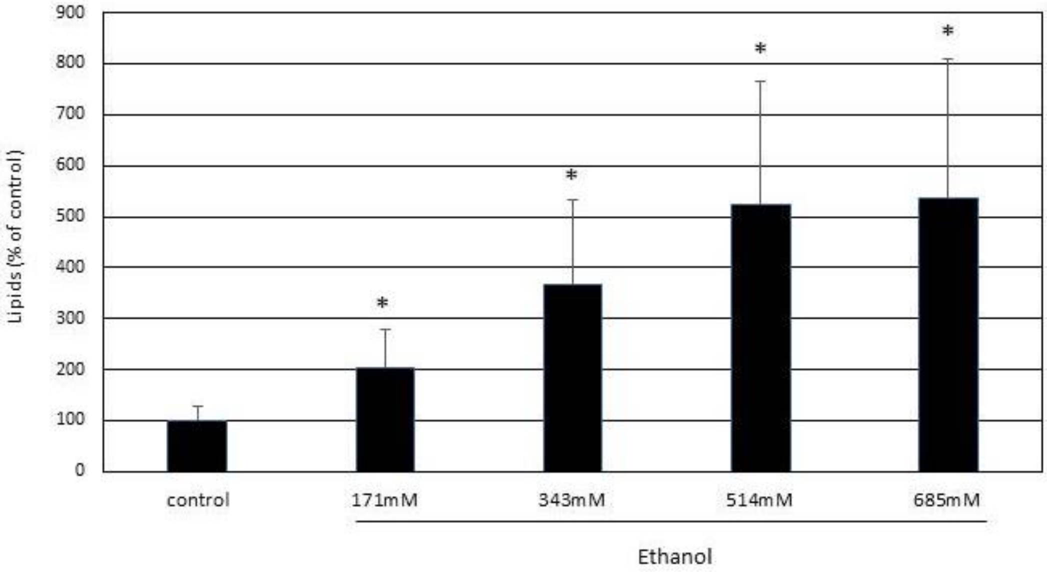


**Fig. S1: Ethanol induces adipogenic differentiation in adipose-derived stem cells (ADSC).** Isolation and initiation of human ADSC cultures were performed as described (Zoller, N., et al. Cells 8:302, 2019). Briefly, subcutaneous fat tissue, derived from abdominal plastic surgeries, generously provided by Dr. Ulrich Rieger (Klinik für Plastische und Ästhetische Chirurgie, Wiederherstellungs- und Handchirurgie, Markus Krankenhaus, Frankfurt/Main, Germany), was placed in PBS with 2% penicillin/streptomycin solution (Biochrom, Berlin, Germany) incubated overnight (4 °C). On the next day, skin and blood vessels were mechanically removed by scissors and forceps. Small pieces, with approximately 5 mm lengths, were given to a collagenase type I solution (Worthington, Lakewood, USA) and incubated for 3 h at 37 °C. Cell debris was discarded by filtration through sterile gauze. Then, the cell suspension was centrifuged (400× g, 6 min, 4 °C), the cell pellet resuspended in medium, and passed through a cell strainer (70 µm, Greiner, Frickenhausen, Germany). Next, cells were separated by density filtration using a Biocoll solution with a specific density of 1.077 g/mL. After another centrifugation (400× g, 30 min, 4 °C), ADSCs were isolated from the opaque interphase and seeded in DMEM supplemented with 1% UltroSerG (Pall, Dreieich, Germany) and 1% penicillin/streptomycin. For the experiment DMEM medium was supplemented with 171, 343, 514 and 685mM ethanol. Cells were allowed to differentiate for 12 days. Then lipid formation was quantified by FACS as described. Each column represents the mean of 45 experiments from 4 different cell isolates. The standard deviations are indicated. Data sets were statistically compared to the controls. *$p<0.05$.
